# Enhancing Arabic healthcare fake news detection with data augmentation and multi-metric analysis using large language models

**DOI:** 10.1038/s41598-025-21733-9

**Published:** 2026-02-09

**Authors:** Ebtsam Mohamed, Walaa N. Ismail, Eman O. Eldawy

**Affiliations:** 1 Faculty of Computers and Information, Minia University, Minia, 61511 Egypt; 2 Department of Management Information Systems, College of Business Administration, Al Yamamah University, Riyadh, 11512 Saudi Arabia

**Keywords:** Large Language Models (LLMs), Arabic Text Generation, Fake News Detection, Healthcare Misinformation, Data Augmentation, Transformer-based Models, Novelty Score, Semantic Similarity, Arabic Data Augmentation, Contextual Embeddings, Health care, Social evolution

## Abstract

The spread of fake news about healthcare can result in a global health crisis, as it is easy to mislead the public. Detection of fake Arabic news in the healthcare sector is crucial for identifying disinformation, especially in regions where Arabic is the predominant language. Various deep learning and machine learning methods have been proposed to categorize false Arabic news related to healthcare. However, the linguistic diversity of Arabic complicates the development of effective models. Furthermore, the lack of domain-specific high-quality data makes it difficult to build accurate and reliable models. Data augmentation (DA) techniques have shown great promise in addressing these challenges. This study presents a novel technique for expanding Arabic healthcare data by conducting a multi-metric analysis to comprehensively evaluate the quality of the augmented data based on several key aspects, including label preservation, novelty, diversity, and semantic similarity. In the initial phase of our research, we investigated the impact of various data augmentation techniques on widely used classification algorithms. Additionally, similarity thresholds are systematically examined to determine their effect on the classification task. Cosine and Jaccard distances are employed to evaluate the generated sentences in terms of semantics, diversity, novelty, and label preservation. Finally, we propose a novel ensemble augmentation approach that combines multiple DA techniques to generate more varied data. Based on the overall experimental results, the proposed methodology significantly improves the classification of Arabic fake news using AraBERT, with an accuracy increase of 12.1%. In comparison, Random Forest achieved an improvement of 14.7%.

## Introduction

Social networks have become increasingly popular. In 2024, more than 5.16 billion people used social networks regularly worldwide, accounting for over 59%. 3% of the population^1,2^. This number is expected to reach six billion users by 2027^3^. The expansion of these platforms, combined with the vast reach of the Internet, has created an environment where information is shared freely, allowing diverse viewpoints to reach audiences at an unprecedented rate. This issue underscores the urgent need for robust mechanisms to verify the credibility of information in an increasingly interconnected digital landscape^4,5^. Promoting public health and ensuring reliable health information necessitates the use of advanced tools, such as algorithms for detecting fake news. These algorithms are crucial in combating misinformation campaigns, supporting evidence-based decision-making, mitigating public anxiety, enhancing health literacy, increasing public trust in healthcare institutions, and fostering effective communication in public health^6,7^. Accurate information is critically essential for safeguarding and improving health outcomes^8^. Machine learning (ML) is a powerful approach for detecting false news in healthcare due to its ability to perform real-time detection, feature learning, automatic detection, scalability, adaptation to changing strategies, and customization to the healthcare sector^5,8^.

According to the American Language Institute^9^,, Arabic is the fifth most commonly spoken language in the world among Semitic languages. This is expected to lead to an increase in digital Arabic content on the Internet^10,11^. Several unique challenges are involved in classifying fake news in Arabic datasets. Some of these challenges are attributed to Arabic’s linguistic and cultural characteristics, while others reflect the more general difficulties associated with detecting fake news. Additionally, the number of large, publicly accessible, high-quality Arabic datasets for false news identification is significantly lower than that of English datasets^10,12^. The lack of labeled data makes training an accurate machine-learning model difficult. Arabic fake news data must be manually classified, a tedious and biased process, particularly when distinguishing between truth and falsehood. The learning model and the knowledge base are key elements of machine learning systems that identify fake news in Arabic. The performance of a learning model is critically dependent on the volume and quality of the training data or knowledge base. Achieving high accuracy requires training on comprehensive datasets, which poses significant challenges in domains such as Arabic fake news detection due to the scarcity of labeled data^10,13,14^.

In the absence of adequate data, regularization approaches are crucial for enhancing model performance. These strategies typically involve data augmentation (DA) techniques or modifying the model’s settings. Data augmentation employs various methods to enlarge and enhance a dataset by making significant changes. The fundamental principle is to increase the quantity, diversity, and representational quality of training data^15–17^.

By incorporating augmented samples into the training dataset, data augmentation reduces overfitting, one of the most common issues encountered when training models on sparse data^18,19^. Furthermore, it generates additional instances of underrepresented classes, which helps address the class imbalance problem often encountered in fake news classification^20^. As a result, DA is a valuable technique for improving the accuracy of machine learning models in detecting fake news in Arabic. Several of these methods are based on the concept of paraphrasing, which can be achieved through the use of transformers, thesaurus searches, or translations^10,18^. A second category of proposed DA strategies involves introducing noise into sentence words using various techniques, such as word swapping, deletion, insertion, and substitution^17,20^. In recent years, large models (LMs) based on the Transformer architecture, such as GPT-2^21^, BERT^22^, T5^23^, large language models^24,25^, and vision models^26^. Time series models^27^, which consist of billions or trillions of parameters and have been pre-trained on massive datasets, offer substantial improvements in automation and diversity in data processing, thereby reducing the need for human intervention^28,29^. Data augmentation is crucial for the robustness and generalization of machine learning models, particularly for large language models (LLMs)^24,30^. Using DA, LLMs can excel in tasks such as processing low-resource languages or analyzing medical text by generating diverse and semantically consistent training examples. Additionally, by introducing controlled variations, DA helps prevent overfitting, enabling LLMs to learn more resilient representations and avoid relying too heavily on spurious patterns^25,29^. This has led to a proliferation of studies focused on industrial intelligence, driven by the growing interest in these capabilities. However, compared to approaches based on English data, there is a noticeable gap in research on using large language models with Arabic data^31–33^. Furthermore, since Arabic differs from other languages, methodologies developed for other languages may not be fully applicable to Arabic textual data^34–36^. Most proposed methods for Arabic rely on conventional data augmentation strategies, such as paraphrasing based on predefined rules and employing noising techniques^10,11^. However, little research has explored methods that significantly enhance the modeling of Arabic textual data through more precise augmentations^12,13^. Methods like Arabic transformers, such as AraBERT and AraGPT2, offer great potential for data augmentation. Additionally, LLMs using transformers can preserve the context of the text^37,38^. This study introduces a novel approach for enriching Arabic textual data by leveraging the capabilities of contemporary large language transformer-based models, specifically AraGPT-2. In the augmentation process, GPT-2 generates additional text samples, enhancing the dataset while maintaining high relevance to the original context and semantics. Several text evaluation criteria are applied to evaluate the quality of the generated sentences. These metrics assess the context, semantics, novelty, and variety of the augmented data using various similarity measures, including cosine and Jaccard distances. The key contributions of this study are as follows:

1. An extensive analysis is conducted to evaluate the impact of various data augmentation techniques on detecting fake news in healthcare. The analysis focuses on the quality of the generated sentences using multiple text evaluation metrics, with an emphasis on label preservation, semantic coherence, diversity, and novelty. Key performance metrics such as F1-score, cosine similarity, BERTScore, and Jaccard scores provide insight into how the augmented data enhances the model’s ability to detect fake news.
2. Similarity thresholds are systematically studied to assess their influence on the classification task, specifically by examining how varying lexical and semantic similarity thresholds affect the model’s ability to classify fake news accurately. These experiments offer insights into the trade-offs between focusing on word-level overlap and meaning-level alignment, as well as how these approaches impact the model’s robustness and generalization capabilities, particularly in detecting nuanced or paraphrased instances of fake news.
3. We propose a novel ensemble data augmentation (DA) approach to overcome the weak performance of large language models, such as GPT, in augmenting Arabic tweets. Our method systematically explores and implements new ensemble augmentation techniques tailored to Arabic text generation. By utilizing GPT’s transformer combined with Synonymous Word Substitution techniques for augmentation, our approach ensures that the generated text maintains semantic integrity and linguistic coherence, which are crucial for handling the complex and morphologically rich Arabic language.
4. Our model incorporates an innovative Arabic text Data augmentation component generated using generative pre-trained transformers (GPT-2). As a result, we can capture subtle linguistic variations and domain-specific vocabulary, enhancing the variety and richness of the training data in the context of Arabic healthcare.

The rest of this paper is organized as follows. Section “Related work” presents an overview of related work. The proposed solution is presented in Sect. "Proposed methodology for Arabic healthcare fake news detection with detection with data augmentation and metric analysis". Section "Experimental results and discussion" presents our experimental results and discussion. Implications for real-world applications are discussed in Sect. "Implication for real-world Application". Finally, Sect. "Conclusion & future work" concludes the paper.

## Related work

Data augmentation is an approach used in machine learning and computer vision to expand the size of a training dataset by generating new, synthetic data from the existing data^39,40^. Data augmentation techniques, such as flipping and rotation, were widely used in the computer vision domain^41^. The Data augmentation goal is to improve the performance and generalizability of machine learning models by exposing them to a diverse range of data variations^39,41^. In DA, the quantity of training data increased by applying different transformations to the training data, thereby developing novel data samples. DA can also increase the variability of the data samples to enhance the model’s performance and prediction accuracy. Furthermore, DA can address the class imbalance problem in classification learning techniques^42^.

### Text augmentation

Recently, DA has been used widely in natural language processing (NLP)^43,44^. Nevertheless, the discrete nature of the data in natural language processing adds more obstacles to smoothly and quickly transforming the input data. That is because the sentence’s meaning may change by varying a word. Thus, more challenging data augmentation techniques begin later in the field of natural language processing^45^. Many DA techniques have been employed in various languages, with the majority being used in English. A few of these techniques augmented the data by rephrasing it using thesauruses^46^, translation^47^, and transformers^48^. Another group of these techniques augmented the text data by adding noise to the sentences, such as deletion^49^, insertion^50^, and swapping words^51^.

Wei and Zou^49^ propose EDA (Easy Data Augmentation) techniques for enhancing the performance of text classification tasks. The EDA exploits text editing techniques for data augmentation. They proposed four straightforward processes: synonym replacement, random insertion, random swap, and random deletion. In^52^, the authors propose a Prompt-based Data Augmentation model (PromDA), which primarily focuses on data augmentation for limited resources in Natural Language Understanding (NLU) tasks. PromDA trains only a set of trainable vectors within frozen Pre-trained Language Models (PLMs). This approach preserves the quality of the synthetic data generated and eliminates the need for human labor to collect unlabeled data within the domain. Furthermore, PromDA generates synthetic data from two distinct perspectives and employs NLU models to filter out lower-quality data.

Back-translation is one of the most widely used data augmentation techniques in NLP, as it is simple to implement and can generate high-quality data. The study of text translation has advanced quickly in recent years. Multiple technology companies, such as Google, have launched translation interfaces. In the Back-translation technique, the data is translated from one language into another and then returned to the original language to generate novel data^53^.

In^54^, the authors study various transformer-based pre-trained models for conditional data augmentation, including auto-regressive models (GPT-2), auto-encoder models (BERT), and seq2seq models (BART). Furthermore, they measure how the data augmentation methods using several pre-trained models differ in data diversity. They also examine how using these methods can preserve the labels of the data.

In^55^, the authors present AugGPT, a ChatGPT-based text data augmentation approach. AugGPT leverages ChatGPT’s capabilities to rephrase sentences in the training data into multiple linguistically distinct yet conceptually comparable ones. After that, AugGPT uses the generated data in a few-shot text classification. They compare the embedding similarity scores between the generated samples and the actual ones to validate the quality and effectiveness of the augmentation method for generating semantically similar variations. However, while AugGPT may be a robust tool for text data augmentation, it can produce incorrect augmentation results due to ChatGPT’s limited domain knowledge in some areas.

Unlike previous work that measures the quality of generated data based only on classification performance, our work measures the quality of generated data through a multi-metric analysis that includes novelty, label preservation, semantic similarity, and diversity. Table 1 compares various text augmentation methods based on their ability to preserve context, diversity, novelty, and the label of the augmented data.

**Table 1:** Comparison of Text Data augmentation techniques.

Research Paper	Augmentation Technique	Semantic Similarity	Measure Diversity	Measure Novelity	Preserve label
EDA: Easy Data Augmentation Techniques for Boosting Performance on Text Classification Tasks^49^	They employed easy data augmentation techniques to improve performance on Text Classification Tasks.	No	No	Yes	No
PromDA: Prompt-based Data Augmentation for Low-Resource NLU Tasks^52^	Constructing synthetic training data utilizing seq2seq Transformer-based PLMs	No	Yes	No	Yes
Multimodal fake news detection through data augmentation-based contrastive learning^39^	A back-translation strategy is used to generate augmented data. Next, they used text and image information that loaded into a BERT-based model to create multimodal features.	Yes	No	Yes	No
AugFake-BERT: Handling Imbalance through Augmentation of Fake News Using BERT to Enhance the Performance of Fake News Classification^56^	Generating artificial fake data by employing Bidirectional Encoder Representation of Transformers (BERT).	Yes	Yes	Yes	No
Data augmentation using pre-trained transformer models^54^	They use various transformer-based pre-trained models for conditional data augmentation, including autoregressive models (GPT-2), autoencoder models (BERT), and seq2seq models (BART).	Yes	Yes	No	Yes
AugGPT: Leveraging ChatGPT for Text Data Augmentation^55^	They exploit ChatGPT for data augmentation	Yes	Yes	No	Yes
Augmentation-Based Ensemble Learning for Stance and Fake News Detection^57^	augmentation-based ensemble learning strategy that combines bagging and stacking.	No	Yes	No	No

### Text augmentation for Arabic language

Despite the reach of the Arabic language, as it is considered the fifth most common language in the world among Semitic languages^11^, few studies have been proposed to augment the Arabic data compared to English^42,58–61^. Moreover, because Arabic has distinguishing characteristics from other languages, not all techniques used for different languages can be applied to Arabic textual data^62^.

Antoun et al.^61^ developed the first Arabic language generation model based on the transformer architecture, ARAGPT2. An enormous collection of filtered Arabic corpora, publicly available, was used to train the model. The perplexity metric, which computes how effectively a probability model predicts a sample, was used to evaluate the model. The results confirm that ARAGPT2 can generate coherent, grammatically sound, and syntactically correct Arabic literature of high quality. In^58^, the authors presented a framework that can augment Arabic phrases. This framework is considered the first to use text augmentation in the Arabic language. Firstly, they labeled the Arabic sentences using human annotators for use in sentiment analysis. This framework could generate new phrases from seed sentences with their correct labels by employing the rich morphology of Arabic, synonymy lists, syntactical or grammatical rules, and negation rules. The words were replaced with their corresponding synonyms using Arabic WordNet^63^. They preserved input sentence labels using these rules.

In^59^, the authors present a data augmentation method for enhancing the results of Named Entity Recognition (NER), particularly for code-switching (CS) in Arabic data. This approach was based on three techniques: back-translation, a modified version of the Easy data augmentation methodology, and word embedding substitution.

Dania et al.^42^ proposed a novel Arabic data augmentation (DA) method that utilizes AraGPT-2, a newly developed powerful modeling technique, to enhance the augmentation process. The augmented phrases are evaluated using Euclidean, cosine, and Jaccard distances for context, semantics, variety, and novelty. Following this, the classification performance of the enhanced Arabic dataset is evaluated using the AraBERT transformer on sentiment classification tasks.

In^19^, the authors present a novel two-stage framework for detecting misinformation in Arabic text. The first stage determines the most effective feature representation before it is entered into the machine learning model. Different representations of tweet content are studied, including N-grams, content-based features, and source-based features. The second stage explores the impact of data augmentation using the back-translation technique applied to the original training data.

Our proposed method differentiates itself from the previous work that focuses explicitly on classification performance. We evaluate the quality of the generated data using a multi-metric analysis, including novelty, label preservation, semantic similarity, and diversity. Table 2 compares various Arabic text augmentation methods based on preserving context, diversity, novelty, and the label of the augmented data.

**Table 2:** Comparison of Arabic Text Data Augmentation Techniques.

Research Paper	Augmentation Technique	Semantic Similarity	Measure Diversity	Measure Novelity	Preserve label
Syntactic- and morphology-based text augmentation framework for Arabic sentiment analysis^58^	A transformation rule-based data augmentation technique for Arabic textual datasets was created after carefully examining Arabic morphology and syntax.	Yes	No	No	Yes
Data augmentation techniques on arabic data for named entity recognition^59^	A technique for data augmentation to enhance Named Entity Recognition (NER) code-switching (CS) results for Arabic data.	Yes	No	No	Yes
Data Augmentation using Transformers and Similarity Measures for Improving Arabic Text Classification^42^	A novel Arabic DA method that uses the AraGPT-2 for enhancing the augmentation process.	Yes	No	Yes	No
A two‑stage framework for Arabic social media text misinformation detection combining data augmentation and AraBERT^19^	A two-stage framework for the automated detection of misinformation in Arabic text.	Yes	No	No	No
Our proposed Technique	A novel approach for enriching Arabic textual data using the capabilities of Contemporary Large Language Transformer-based models (LLMs) for both augmentation and classification tasks	Yes	Yes	Yes	Yes

### Text augmentation for fake news

In an era of rapidly growing information, news can be systematically manipulated to spread social influence and generate misinformation, including fake news. Dishonestly propagating fake news has destructive effects, such as leading to poor decisions^64^. Hua et al.^64^ proposed a BERT-based back-translation Text and entire image multimodal machine learning-based framework that combines contrastive learning with text translation (TTEC). In the TTEC approach, a multi-head attention method is used to extract relevant features from multimodal data, addressing combined information that involves text and images^64^. To resolve the minority problem of the class, the AugFake-BERT model is suggested^56^. In the AugFake-BERT model, they create an augmented dataset consisting of artificially created fake data. Then, they represent a text augmentation technique using a Bidirectional Encoder Representation of Transformers (BERT) language model. In^57^, they explore how text data augmentation techniques can be used to identify stances and detect fake news. First, they study how the performance of the standard classification algorithms can be affected by using different text augmentation methods. Additionally, they identify the optimal pairs of classification algorithms and augmentation methods that work together to achieve the highest accuracy. Second, they suggest an augmentation-based ensemble learning strategy that combines bagging and stacking techniques. This method used text augmentation to enhance the accuracy and diversity of base learners, which in turn improves the ensemble’s predictive performance.

### Comparison with related literature

The lack of data challenges modern techniques for identifying fake news about Arabic healthcare. According to Table 3, research gaps in Arabic healthcare fake news detection are summarized and categorized, along with flaws in current models based on recent publications. In contrast to previous related work, the proposed model proposes an Arabic text enrichment procedure that utilizes large Arabic models to mitigate this problem. Our study improves the precision and dependability of Healthcare data analysis by methodically addressing these gaps.

**Table 3:** Research gaps in existing models.

Research Gap	Augmentation Technique
Insufficient Data Augmentation Diversity	In most current models, only one data augmentation strategy is used, ignoring the advantages of combining several techniques to enhance the diversity and resilience of the training data.
Effect of Varying Semantic Similarity on Performance	Current models rarely examine how changes in the semantic similarity between enhanced and original sentences affect classification performance, especially in terms of model generalization.
Balancing Label Preservation and Content Enrichment	Existing models fail to consider the need to strike a balance between adding new content to augmented sentences and maintaining their original class labels and meanings.
Arabic Subtle Linguistic Variations	The performance of current Arabic language models is generally unsatisfactory when applied to texts that do not conform to the Modern Standard Arabic (MSA).
A standard limitation of current methods is their reliance on conventional evaluation criteria, such as accuracy, while ignoring other important aspects, including the semantic variance, diversity, and novelty of new augmented data, and how these factors may impact model performance.	

## Proposed methodology for Arabic healthcare fake news detection with detection with data augmentation and metric analysis

**Fig. 1:**
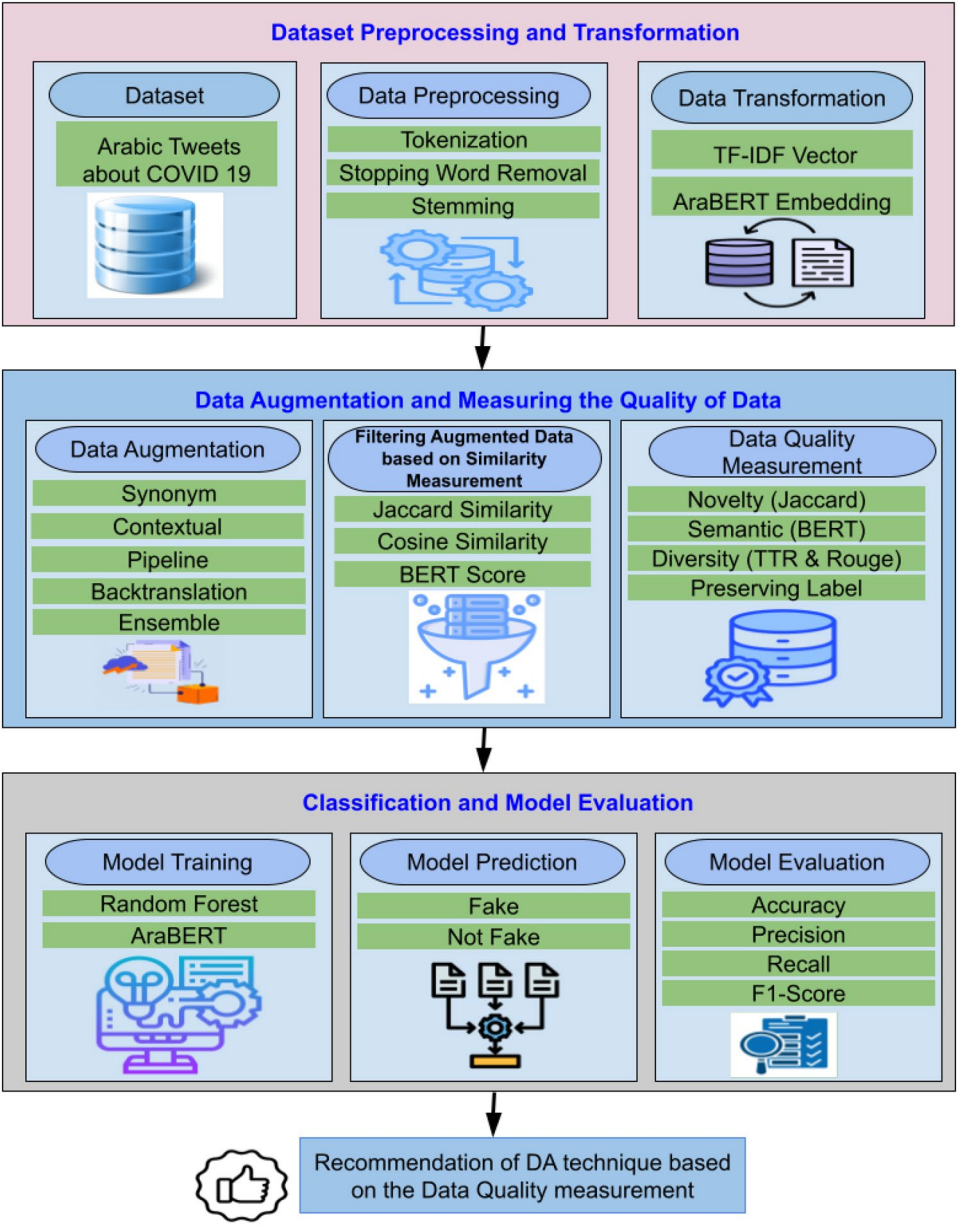
Working steps of the proposed Arabic Healthcare Fake News Detection.

In this section, a fake news detection system is proposed for the healthcare industry that can adapt to evolving trends in misinformation. The proposed classification approach is shown in Fig. 1. Preparing the data is the first step in the augmentation process. Raw text data is cleaned and standardized during this phase to ensure consistency. The process involves removing extraneous characters from Arabic texts, including punctuation and special symbols, as well as managing stop words and diacritical marks. Once preprocessing is completed, various text augmentation techniques are applied, which affect the preservation of class labels, semantics, diversity, and novelty in the generated sentences. Extensive experiments are conducted to identify the most accurate combinations of augmentation techniques and classification algorithms. Evaluation metrics—including Type-Token Ratio (TTR)^65^, cosine similarity^66^, BERTScore^67^, and Jaccard similarity scores^68^—are assessed. Subsequently, the system generates a new dataset containing the augmented Arabic text. It then trains a model on this augmented dataset to classify news as real or fake. The model’s performance is evaluated using various metrics, and the system is implemented for practical use.

The suggested model incorporates context analysis to assess both the reliability of sources and the semantic content of Arabic-generated text. To integrate sentence context into the proposed model, changes in label semantics, diversity, and novelty—key properties of fake and authentic healthcare news stories—are tracked over time. This work enhances the performance of fake news detection systems in Arabic. It contributes to the broader field of Arabic natural language processing by providing a robust data augmentation and model training framework.

### Dataset pre-processing and transformation

As the Arabic language has considerable morphological richness - a single Arabic word may have many meanings and forms - a thorough understanding of the language is essential. Our suggested technique has been tested and applied to a fake news dataset in Arabic, as shown in Table 4. The dataset used and preprocessing tasks are described below:

**Dataset:** This research used Arabic healthcare data on infectious diseases from September 2019 to focus on COVID-19 tweets. Based on three primary keywords related to the infection^69^. In the original dataset, approximately six million tweets were collected. A Twitter API was used to collect tweets on a weekly basis. After collecting the data, the authors manually eliminated advertisements, spam, and retweets. Furthermore, Python scripts filter out words, digits, emoticons, hashtags, URLs, and mentions not in Arabic. According to the paper’s primary author, each tweet was categorized into one of five categories^69^: Academic, Media, Healthcare Professional, Government, and Public. The primary focus of this study is to classify and evaluate tweets related to healthcare, after normalizing and tokenizing the text and removing Arabic stopwords. The Saudi Ministry of Health’s official list^70^ updated daily served as the foundation for labeling tweets as true, false, or irrelevant. The labels for the tweets, representing accurate information, unrelated material, and incorrect information, are 1, −1, and 0, respectively. The dataset was manually annotated for only 2,365 tweets, while the remaining tweets were annotated automatically. To obtain more robust results, we conducted our experiments using only the manually annotated labels, which consist of 2,365 tagged tweets, 30,637 words, and 7,798 unique words. Table 4 presents the statistics of the original dataset.

**Table 4:** The statistics of original dataset.

No. of Tweets	Fake Tweets	Non-Fake Tweets
Tweets	1284	1081
Words	30,637	30,075
Unique Words	7,798	8,706

**Data Preprocessing:** This step cleans and standardizes raw text data to ensure consistency and accuracy. The process involves removing extraneous characters from Arabic texts, including punctuation and special symbols, as well as managing stop words and diacritical marks. Lemmatization and stemming also reduce words to their simplest forms, similar to tokenization.

**Data Transformation**: To convert raw text into meaningful numerical representations, TF-IDF (Term Frequency - Inverse Document Frequency)^71^ is applied with Random Forest (RF) algorithm^72^. TF-IDF is a powerful feature extraction technique, especially for traditional machine learning models, such as RF. However, our ARaBERT model utilizes word embeddings (WE) to capture semantic information and relationships between words^73^.

### Data augmentation and measuring the quality of data

This stage is used to augment new Arabic healthcare tweets from the existing ones. After that, we filter these augmented data based on the similarity to improve its quality (as shown in Fig. 1). Different data augmentation techniques are applied. At this stage, the generated data quality is measured by several criteria, including preserving the label, semantics, novelty, and diversity for different data augmentation (DA) techniques. These criteria are discussed as follows.

**Label Preservation**: A method to ensure that the augmented instance retains the same class label as the original data point, which is crucial for effective model training. Failure to preserve the label can introduce noise, potentially misleading the model during training. Textual DA techniques, such as back-translation and synonym substitution, help maintain label integrity by ensuring the modified sentence remains in its correct category.

**Preserving Semantics:** Augmented data should introduce variety while preserving the original content’s meaning and context. To achieve this, back-translation and transformer-based models (e.g., BART, T5) generate rephrased sentences while preserving the original intent.

**Novelty:** Novelty refers to the degree to which augmented data differs from the original instance while remaining valid. Higher novelty exposes the model to a wider range of patterns, improving its adaptability.

**Diversity:** Diversity represents the extent of variation introduced through augmentation across different samples. Ensuring diversity prevents overfitting and enhances generalization across unseen data distributions.

**Fig. 2:**
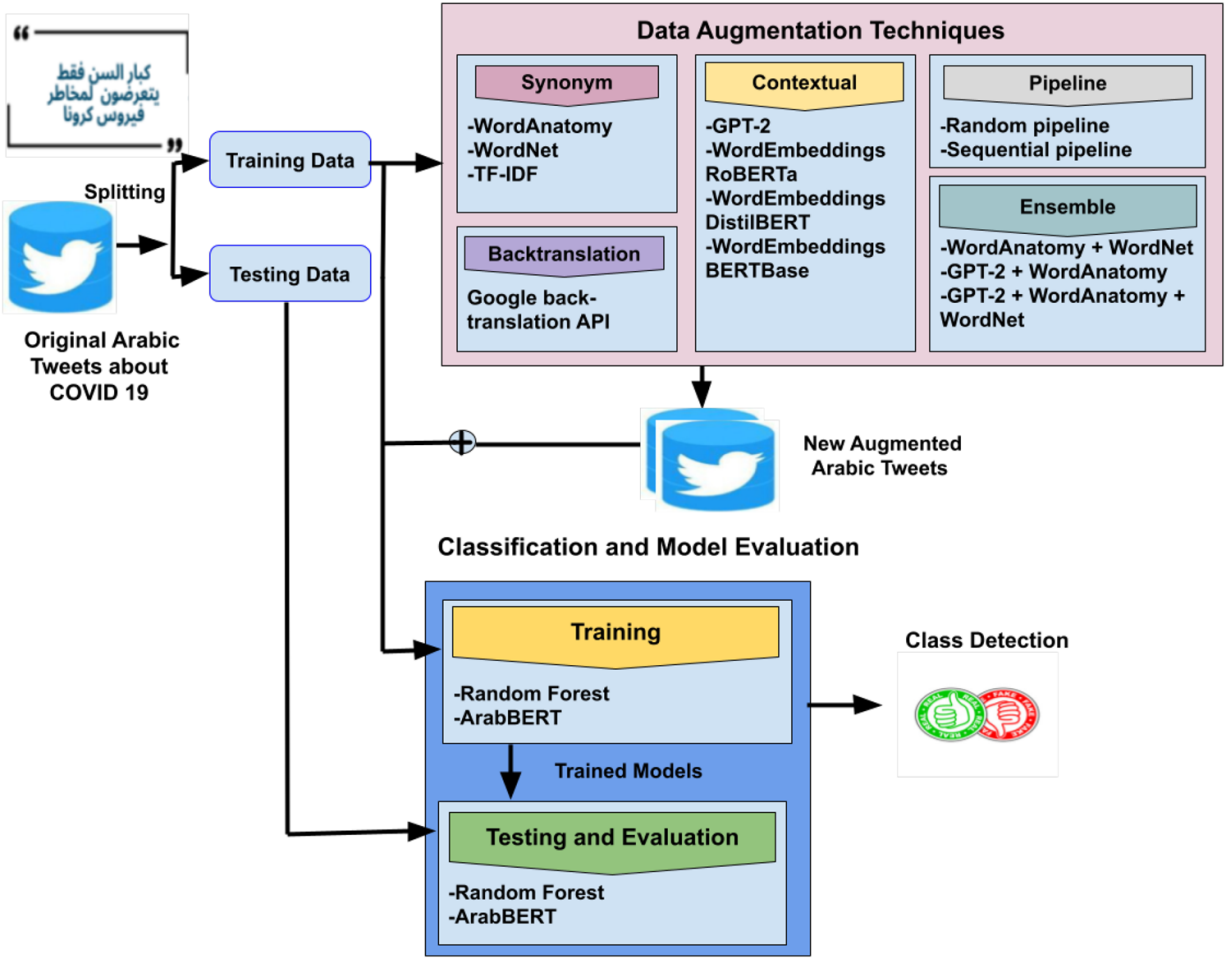
Augmentation workflow for Arabic healthcare.

**Algorithm 1:**
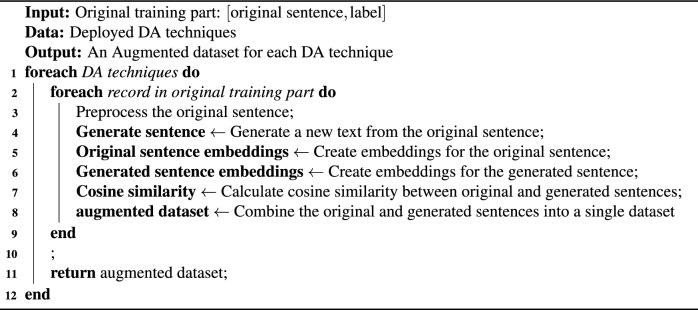
Arabic Text Data Generation

The data augmentation process, as shown in Algorithm 1 and Fig. 2, begins by separating the original dataset into a 80% training set and a 20% test set. The augmentation process removes duplicate tweets to ensure data quality and prevent overfitting.

#### Step 1: data augmentation

This study used data augmentation for Arabic to increase the variability and diversity of the training dataset. Data augmentation involves modifying the initial training data to obtain fresh, synthetic samples. For example, in synonym replacement, the sentence: “The weather today is beautiful. This could be augmented by replacing “beautiful” with its synonym “wonderful,” resulting in: “The weather today is wonderful.” The test set was selected independently of the enhanced training data to assess the model’s performance objectively. The study conducted trials using the same test set derived from the original dataset. Consequently, the evaluation is free from bias caused by the data augmentation procedure and accurately represents the impact of augmentation strategies on model performance.

A rigorous evaluation of different metrics (including Algorithm 2) is implemented to determine the effectiveness of DA techniques in increasing data diversity, preserving labels, and consequently improving model performance. Moreover, the current study examined optimal similarity thresholds to balance the trade-off between diversity and relevance in the augmented data.

**Fig. 3:**
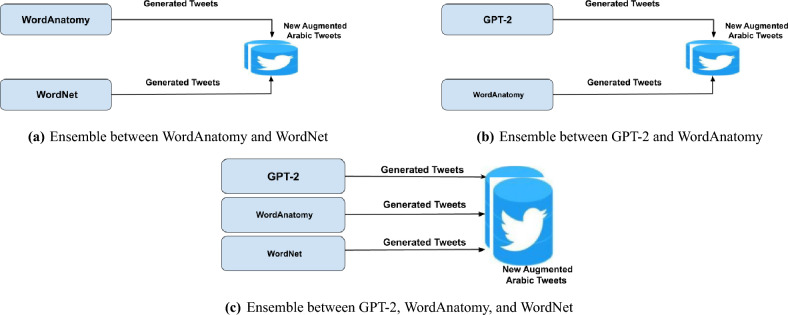
Ensemble Data Augementation Techniques.

In this paper, we present five DA techniques that are summarized as follows:

1. **Synonym (Word substitution)**: Word substitution replaces words in a sentence with their synonyms while maintaining the meaning of the original sentence. Using this technique can generate new variations of the text to augment data. We used WordAntonym Substitution, WordNet, and TF-IDF in our proposed approach. Despite their topical similarity, the original and generated texts often diverge significantly in factual alignment, mainly when using the antonym replacement technique. Sometimes, the generated text conveys the opposite meaning while retaining the same class label. As a result, the model frequently generates unexpected words and misleading examples. The system reverses the class label to ensure consistency between the generated and original text. For example, in the original text, “Asian workers, most of them dirty, spit everywhere, we do not want them to,” the corresponding generated text is, “Asian workers, fewest of them dirty, spit everywhere, we do not want them to.” where the word “most” is replaced by “fewest.” In such cases, the class label is reversed from ’fake’ to ’real’ or vice versa to maintain the factual meaning unchanged.
2. **Backtranslation**: a text is translated into another language and then returned to the original language, introducing subtle yet significant changes. The back translation process enables the model to view new text versions that retain the original message but differ in structure or word choice. This method is beneficial in exposing models to a variety of linguistic forms that may appear in fake news. In our proposed approach, we used the Google backtranslation API.
3. **Contextual using Transformers**: Using a transformer, models can manage long-range dependencies and simultaneously calculate the relationships between every word in a phrase. Pre-trained transformers, such as BERT and RoBERTa, demonstrate high effectiveness at generalizing across tasks, as pretraining equips these models with a profound understanding of the task. Our approach utilizes GPT-2, WordEmbeddings RoBERTa, WordEmbeddings DistilBERT, and WordEmbeddings BERTBase, with several reasons for selecting GPT-2 for this investigation. As a first step, this study focuses on Arabic data augmentation to create representative and diverse datasets. GPT-2 provides sufficient capacity to meet task requirements without requiring the extra complexity and resources of more sophisticated models, such as GPT-4. In this way, performance and methodology can be evaluated from an efficient starting point, providing valuable information for comparisons with larger and more complex models in the future. Furthermore, practical computational and resource efficiency considerations were taken into account when selecting GPT-2.
4. **Pipeline**: In the pipeline technique, a group of augmentation functions is selected to be applied to the original tweet sequentially or randomly. In our technique, three functions are used. The first function is RandomWordAug, which randomly swaps, crops, or deletes words. The second function is AntonymAug, which substitutes a word with an opposite meaning based on WordNet. The last function is ContextualWordEmbsAug, which identifies the most suitable word for substitution using the BERT or RoBERT model. The pipeline sequential technique applies the three functions sequentially, where the output of one function is fed as input to the next. The final augmented tweet is the output of the last function in the sequence. However, in the Pipeline_random technique, the functions are applied randomly.
5. **Ensemble**: A novel ensemble DA approach is proposed to enhance the performance of GPT large language models for augmentation of the Arabic tweets. Our method systematically investigates and implements new ensemble augmentation techniques for generating Arabic text. Combining GPT’s transformer with Synonymous Word Substitution techniques in the augmentation process can generate text that maintains semantic integrity and linguistic coherence. We combine WordAnatomy and WordNet in our ensemble approach, as described in Fig. 3a. Moreover, we combine GPT-2 and WordAnatomy, as displayed in Fig. 3b. Additionally, we combine GPT-2, WordAnatomy, and WordNet, as illustrated in Fig. 3c. The combinations mean adding generated instances from each DA technique as one dataset without duplication. This addition provides variation in the distributed terms in the dataset. Three task-specific transformation functions are combined:

#### Step 2: filtering augmented data based on similarity measurement

As part of the refinement process, the augmented data is filtered based on Jaccard, BERT score, and cosine similarity. Tweets with a similarity score of 0.5 or higher are retained, resulting in a filtered dataset. We perform a grid search across BERTScore and Cosine Similarity thresholds in the range [0.6, 0.7, 0.8, 0.9], selecting the threshold that produces the best model performance. This filtered dataset is then used to retrain the model, and the results are re-evaluated using the original dataset. Using this multi-step augmentation and filtering process, one can assess the impact of data augmentation on model performance while ensuring semantic relevance.

**Algorithm 2:**
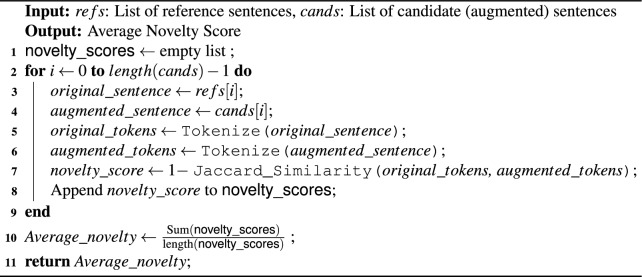
Calculate Novelty Scores

#### Step 3: data quality measurement

The last step in the augmentation process is evaluating the quality of the generated sentences. In our framework, several text evaluation criteria are used to measure the context, semantics, novelty, and variety of enhanced data. The semantic alignment between the generated and original sentences is measured using BERTScore and cosine similarity to ensure the enhanced data maintains its original meaning. To encourage heterogeneity within the dataset, the Type-Token Ratio and Rouge similarity^74^ are used to evaluate the diversity of the created information. Jaccard similarity ensures the novelty of the augmented text at the word level. This combination optimizes the augmentation process and enhances the model’s ability to identify false news.

### Classification and model evaluation

The primary goal of this study is to retrain the classification models on the augmented new datasets (the output of stage 2) and evaluate their effectiveness in detecting false news in Arabic text. We use hold-out cross-validation, where the dataset is split into 80% training and 20% testing sets. We also use 5-fold cross-validation in experiment A to check model overfitting.

Firstly, when training models, we train them on the training data and the new augmented Arabic tweets. AraBERT and RF are used to classify Arabic text data. Performance indicators, such as accuracy, precision, Recall, and F1-score, are used to assess the effectiveness of the ensemble model.

After training the models, their accuracy is tested using the testing data. AraBERT and RF are used to classify the testing Arabic text data. The output is classifications of fake news. Finally, the model’s performance is measured using evaluation metrics. We use many evaluation metrics, such as accuracy, precision, Recall, and F1 score (the macro average value is used in this paper), to measure the performance of the models. At the end of our framework, we output a recommendation for the data augmentation technique based on the data quality measurement provided by our framework.

## Experimental results and discussion

Augmentation techniques are applied to generate new tweets based on the training data. Generated tweets retain the same labels as their corresponding original tweets. To establish a baseline for the model, we evaluate its performance on the original training part using the unaltered test set. The augmented tweets are then added to the original training data, resulting in a non-filtered dataset. Following this, the model is retrained using the new augmented dataset, and the results are compared with the original dataset. To enhance text data processing and augmentation, the proposed implementation integrates the NLP augmentation library from^75^. The paper presents several experiments, each of which has a different objective as follows:

- **Experiment A:** Analyzing the effects of augmentation on the classification performance of fake news. RF and AraBERT classifiers are applied to the original and augmented datasets.
- **Experiment B:** Determining and establishing the best threshold for the similarity between the original Arabic tweet and the augmented tweet. The augmented dataset is filtered according to threshold values for cosine similarity and BERTScore measures. To evaluate the effect of contextual augmentation, we compared the classification results with those obtained using cosine and score similarity measures.
- **Experiment C:** Evaluating data quality of the suggested augmentation methods regarding label preserving, novelty, diversity, and semantics. Additionally, determine whether the proposed methods consider the context of the augmentation process.
- **Experiment D:** Evaluating the effect of a proposed ensemble augmentation approach on Arabic Healthcare classification performance.
- Comparison between our proposed approach and the previous approaches that use the same dataset.

### Experiment A: experiment assess the effectiveness of the augmentation process in identifying fake Arabic news

This experiment evaluates two classification models, RF and AraBERT, based on augmented datasets that have been subjected to various augmentation techniques. The similarity score is ignored when merging all newly created data with the original dataset. To ensure a fair comparison between different DA techniques, the same number of augmented tweets (1,203 tweets) was generated and used in this experiment.

Table 5 displays the categorization results for the original dataset, which serve as our baseline results. In Tables 6 and 7, the results of the RF and AraBERT classifiers (using original $+$ new augmented tweets) are presented. Eight augmentation techniques (WordAntonym, WordNet, TFIDF, WordEmbeddings _BERTBase, WordEmbeddings_DistilBERT, WordEmbeddings_RoBERTa, pipline_random, and pipline_sequential techniques) are applied to the original dataset to provide a more comprehensive analysis. The augmentation process adds the same number of instances for each class as newly generated tweets to the original dataset to keep the dataset balanced.

Table 6 shows the results of RF for the Not-Filtered Dataset. We can observe that RF’s performance improves significantly with augmentation, especially with the WordAntonym method, surpassing results from the original dataset. The highest performance across all metrics was achieved by WordAntonym, with accuracies of 92.45%, precision of 93%, recall of 92%, and an F-score of 92%. The TF-IDF method performed the least well out of the four methods, with an accuracy of 87.17%, precision, Recall, and F-score of 87%. In Table 7, the results of the AraBERT model for the Not-Filtered Dataset are displayed. It is observed that the WordNet DA method achieves higher accuracy compared to other methods.

For both RF and AraBERT classifiers, the performance of the augmented dataset is better than that of the original dataset, suggesting that augmentation may be a valuable means of improving the classification performance.

**Table 5:** Results of Original Dataset.

Model	Accuracy(%)	Prec.(%)	Recall.(%)	F-score(%)
RF	79.62	80	80	80
AraBERT	83.73	83	84	84

**Table 6:** Results RF of Not-Filtered Dataset (with no similarity).

Method	Accuracy(%)	Prec.(%)	Recall.(%)	F-score(%)
WordAntonym	92.45	93	92	92
WordNet	90.19	90	90	90
TF-IDF	87.17	88	87	87
WordEmbeddings_BERTBase	79.25	80	79	79
WordEmbeddings _DistilBERT	79.25	80	79	79
WordEmbeddings _RoBERTa	78.49	79	78	78
Pipeline_random	75.84	77	76	75
Pipeline_sequential	76.98	78	77	77

**Table 7:** Results AraBERT of Not-Filtered Dataset (with no similarity).

Method	Accuracy(%)	Prec.(%)	Recall.(%)	F-score(%)
WordAntonym	95.13	95	94	95
WordNet	95.81	96	95	95
TF-IDF	93.40	94	93	93
WordEmbeddings_BERTBase	85.80	85	86	85
WordEmbeddings _DistilBERT	84.72	85	84	85
WordEmbeddings _RoBERTa	84.32	84	84	84
Pipeline_random	81.54	81	81	81
Pipeline_sequential	82.61	82	83	82

**Table 8:** Performance metrics for 5-fold cross-validation of RF classifier.

Metric	Without Augmentation	With Augmentation
Accuracy (%) per Fold		
Fold 1	79.54	93.56
Fold 2	78.78	94.69
Fold 3	79.54	92.80
Fold 4	75.00	91.28
Fold 5	79.16	94.31
**Average Accuracy**	**78.40**	**93.33**
Average Precision, Recall, and F-score (%)		
Precision	78.60	93.40
Recall	78.40	93.20
F-score	78.60	93.40

**Fig. 4:**
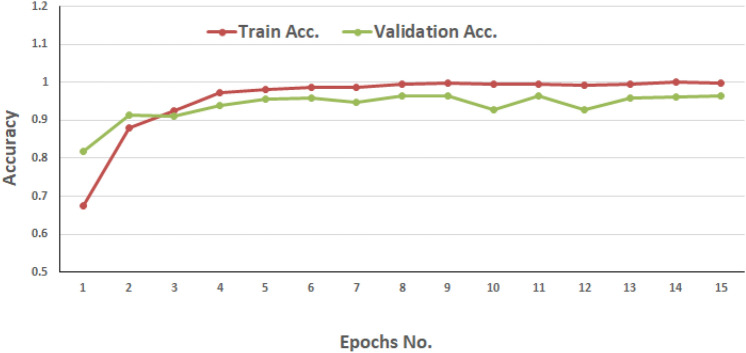
AraBERT Performance Evaluation using Augmented Dataset.

According to Tables 6 and 7, the AraBERT classifier outperforms the RF model for all enhancement strategies. The AraBERT model outperforms the RF model by 2.68% for WordAntonym and 5.62% for WordNet. Additionally, while both models receive the same number of tweets through augmentation, AraBERT uses the augmented data more effectively, achieving a higher score.

Additional experiments were conducted for the RF and AraBERT models to detect potential model overfitting. The RF model was retrained using 5-fold cross-validation on both the original dataset and an augmented dataset containing generated tweets, as presented in Table 8. For the AraBERT model, we ran 15 epochs on the original dataset after adding the augmented tweets. Figure 4 illustrates the training and validation accuracy over the 15 epochs.

Based on the performance across all folds, as illustrated in Table 8 and Fig. 4, the augmentation technique improves model generalization without introducing biases or redundancies. The results indicate that the RF model does not exhibit overfitting during training, and the test results are robust and reliable. Additionally, the training and validation accuracy of the AraBert model improved throughout the epochs, indicating that the model continued to perform well after the inclusion of augmented tweets.

Overall, the replacement techniques (WordAntonym, WordNet) perform better than others. This is because they do not alter the context of the input tweet; instead, they replace words with related terms or synonyms from the WordNet dictionary. In contrast, contextual word embedding techniques achieve lower performance in classification tasks. These techniques rely on pre-trained deep-learning models to replace words in the original tweets based on contextual understanding. However, since most tweets are short, they do not provide sufficient context for these models. Additionally, their performance depends on the size and domain of the corpus used for training. As a result, these models often generate tweets with different contexts and lower similarity to the original ones. Pipeline-based augmentation was tested but proved to be significantly less effective than alternative augmentation methods, with a maximum accuracy of 82.61%. Consequently, it was excluded from subsequent analyses to focus on more effective techniques that yielded better results.

### Experiment B: the impact of similarity threshold on classification performance

In this section, a systematic analysis is conducted to assess the impact of filtering on classification outcomes and the model’s overall effectiveness. Preliminary research suggests that generating data with a higher similarity threshold could minimize noise and thus improve precision at the expense of Recall. However, low similarity requirements generally allow for more sentence changes. As a result, Recall may be enhanced, but accuracy may be compromised as duplicate or irrelevant data is added. A balance will be maintained between these measures, emphasizing the trade-offs of choosing a similarity threshold and its impact on the models’ long-term classification. According to the results of Experiment A, we selected two DA techniques with the best performance, namely Word_Antonym and WordNet, to investigate the impact of their similarity on augmentation performance. Table 9 displays the RF results of the filtered BERT similarity dataset of the generated tweets utilizing the Word_Antonym method. As is clear in Table 9, the threshold range (0.4–1.0) outperforms others as it generates more tweets. Additionally, Table 10 presents the RF results of the filtered BERT similarity dataset of the generated tweets of the WordNet technique. Also, the threshold range (0.4–1.0) outperforms others and generates the highest number of tweets compared to the other threshold ranges.

In Table 11, the results of the AraBERT model of the filtered BERT similarity dataset of the generated tweets by using the $Word\_Antonym$ method are presented. AraBERT’s accuracy level of 97.22% is achieved using $Word\_Antonym$ augmentation for the range $0.5 \le \text {Score} \le 1.0$, which is the best performer and the ideal range for accurate forecasts. This range comprises the most tweets (1125). While AraBERT, with high similarity scores (greater than 0.8), achieves an accuracy of 87.85%, this indicates a decline in rewards. Additionally, Table 12 presents the results of the AraBERT model on the filtered BERT similarity dataset of generated tweets, obtained using the WordNet method. Furthermore, the threshold range (0.4–1.0) surpasses others and generates more tweets compared to the other threshold ranges.

**Table 9:** RF Results of Filtered-based BERT Similarity Dataset (Word_Antonym).

Threshold Range	Accuracy(%)	Prec.(%)	Recall(%)	F-score(%)	New Tweets
0.4–1	93.58	94	94	94	1125
0.5–1	92.83	93	93	93	1112
0.6–1	89.81	90	90	90	1093
0.7–1	86.03	87	86	86	1050
0.8–1	83.77	84	84	84	956
0.9–1	80.75	81	81	81	707

**Table 10:** RF Results of Filtered Dataset Based on BERT-Similarity Score (WordNet).

Threshold Range	Accuracy(%)	Prec.(%)	Recall(%)	F-score(%)	New Tweets
0.4–1	90.94	91	91	91	1197
0.5–1	89.43	90	89	89	1166
0.6–1	86.41	87	86	86	1094
0.7–1	82.26	83	82	82	955
0.8–1	79.24	80	79	79	736
0.9–1	81.51	82	82	81	365

**Table 11:** AraBERT Results of Filtered-based BERT similarity Dataset (Word_Antonym).

Threshold Range	Accuracy(%)	Prec.(%)	Recall(%)	F-score(%)	New Tweets
0.4–1	96.87	96.90	96.73	96.82	1125
0.5–1.5	97.22	97.12	97.25	97.15	1112
0.6–1	95.49	95.53	95.45	95.50	1093
0.7–1	90.63	91	90	91	1050
0.8–1	87.58	87.91	87.86	87.87	956
0.9–1	86.80	87.13	86.32	86.82	707

**Table 12:** AraBERT Results of Filtered Dataset Based on BERT-Similarity Score (WordNet).

Threshold Range	Accuracy(%)	Prec.(%)	Recall(%)	F-score(%)	New Tweets
0.4–1	93.10	93	94	93	1197
0.5–1	91.12	91	92	91	1166
0.6–1	89.23	89	90	89	1094
0.7–1	87.25	87	88	87	955
0.8–1	84.45	84	85	84	736
0.9–1	85.42	85	86	85	365

From Tables 9, 10, 11, 12 we can observe that a balance between similarity and growth (number of tweets) of generated data is associated with higher classification performance. The classification task at specific similarity ranges (0.4–1.0 and 0.5–1.0) outperforms the others. The reason is that newly generated sentences have no excessive similarity with the original corpus within the same class label. As shown in experiments A and B, the RF model improved from 92.45% without similarity classification to 93.58% after similarity classification. Finally, we can conclude that tweet volume and accuracy provide a strategic direction for refining models to optimize performance and data utility. In general, similarity-based filtering can benefit both RF and AraBERT models.

### Experiment C: evaluating the data quality of augmentation techniques in terms of preserving label, semantics, novelty, and diversity

Through experiment C, our goal was to evaluate the quality of sentences generated by the different data augmentation techniques. This evaluation employs a combination of metrics, including the ability to preserve class labels, semantics, diversity, and novelty.

**Table 13:** RF Results of Filtered Dataset based on Cosine Similarity Score (Word_Antonym).

Threshold Range	Accuracy(%)	Prec.(%)	Recall(%)	F-score(%)	New Tweets
0.4–1	79.62	80	80	79	967
0.6–1	81.13	82	81	81	927
0.8–1	80.37	81	80	80	666

**Table 14:** RF Results of Filtered Dataset based on Cosine-Similarity Score (WordNet).

Threshold Range	Accuracy(%)	Prec.(%)	Recall(%)	F-score(%)	New Tweets
0.4–1	78.87	80	79	79	1040
0.6–1	80.00	80	80	80	954
0.8–1	78.49	79	78	78	534

**Fig. 5:**
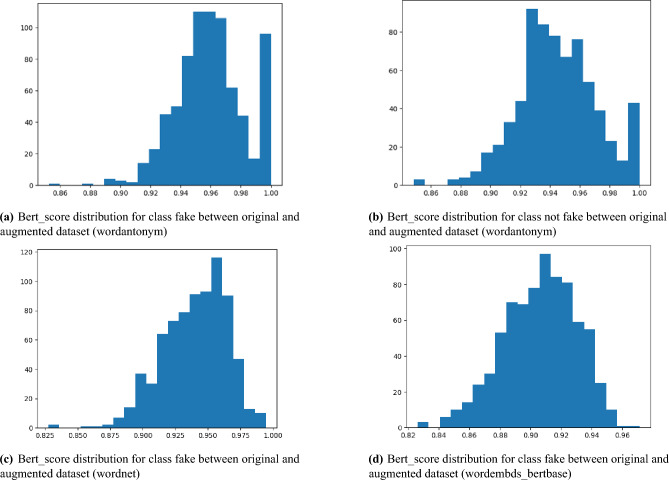
BERTscore Similarity Score Distribution.

To assess the ability of the DA technique to preserve semantics, we compare the classification results based on cosine similarity (text match) with those based on BERTScore similarity (semantic match). So filtering process is conducted based on cosine similarity as shown in Tables 13 and 14. Table 13 presents the classification results when filtering the augmented sentences produced by word-antonym. With increasing similarity thresholds, classification performance has steadily improved, reaching a peak around the 0.6–1.0 range (Accuracy: 81.13%, F1-score: 81%). Additionally, Table 14, illustrates text classification results using augmented data produced by WordNet and filtered by cosine similarity thresholds. Achieving 80% accuracy and F1-score while utilizing mid-range similarity criteria (0.6–1.0) increases classification performance, indicating that enhanced sentences within this range are more useful for the model because they are semantically similar to the original sentences. Performance slightly declines at the higher threshold (0.8–1.0) (Accuracy: 78.09%, F1: 78%), as excessive constraints restrict diversity and lose augmentation gains.

Based on the results of both tables, the semantic quality control using cosine similarity enhances the efficacy of data augmentation. However, there is a trade-off between the quantity of the dataset and semantic purity. As a result of the ideal augmentation, which occurs at a moderate similarity threshold (0.6–1.0), the model achieves both performance and generalization. According to these findings, sentence-level augmentation necessitates the careful adjustment of similarity filters to strike a balance between the quantity and quality of the data.

By comparing Tables 9 and 10 with Tables 13 and 14, a comparative analysis demonstrates that BERT similarity outperforms cosine similarity in preserving semantic integrity during augmentation. WordNetbased augmentation accuracy declined from 90.94% to 80% under cosine similarity filtering, while Word_Antonymbased performance dropped from 93.58% to 81.13%, exhibiting BERT superior effectiveness in maintaining semantic. This superiority of BERT similarity leads to improved classification performance.

Additionally, providing the similarity between the original and augmented tweets based on BERTScores as illustrated in Fig. 5. Figure 5 demonstrates the similarity between the original and augmented tweets based on BERTScores. As indicated by the x-axis of the histogram, the BERTScore values show the semantic similarity between the original and enhanced text. On the y-axis, the frequency of each BERTScore value appears, representing how often each similarity level occurs in the “fake” class samples from both the original and augmented datasets. By using BERTScore (Fig. 5a through 5d), we demonstrate how context-aware evaluation provides a more nuanced understanding of text similarity. This approach better captures the semantic relationships within the text, thus improving the classification model’s overall performance.

**Fig. 6:**
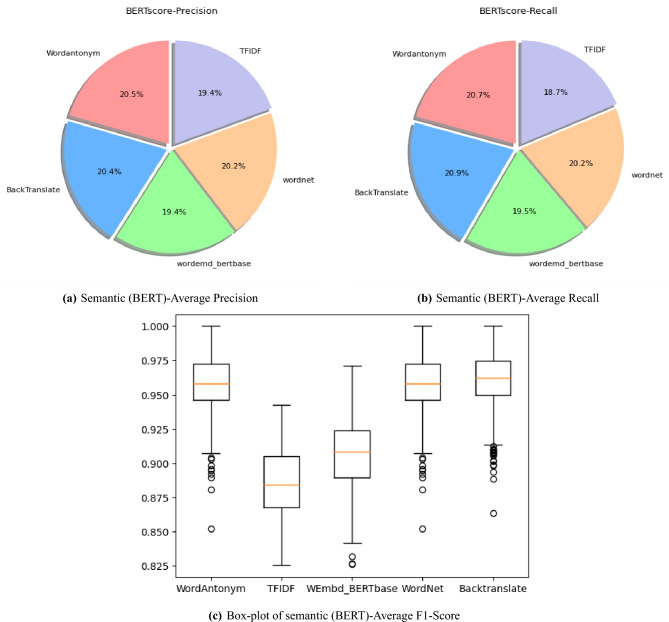
Comparison of semantic (BERT) Precision, Recall, and F1-Score for Different Augmentation Techniques.

To assess the ability of the DA technique to provide novel data, the average novelty score is calculated for different augmented datasets using the Jaccard similarity metric, as shown in Fig. 6. In text augmentation, it is crucial to strike a balance between semantic similarity and semantic novelty. Higher semantic novelty scores (closer to 1) generally reflect a more significant semantic difference between the original and augmented text. The threshold for what constitutes “significant” novelty varies depending on the specific task and desired level of augmentation.

**Fig. 7:**
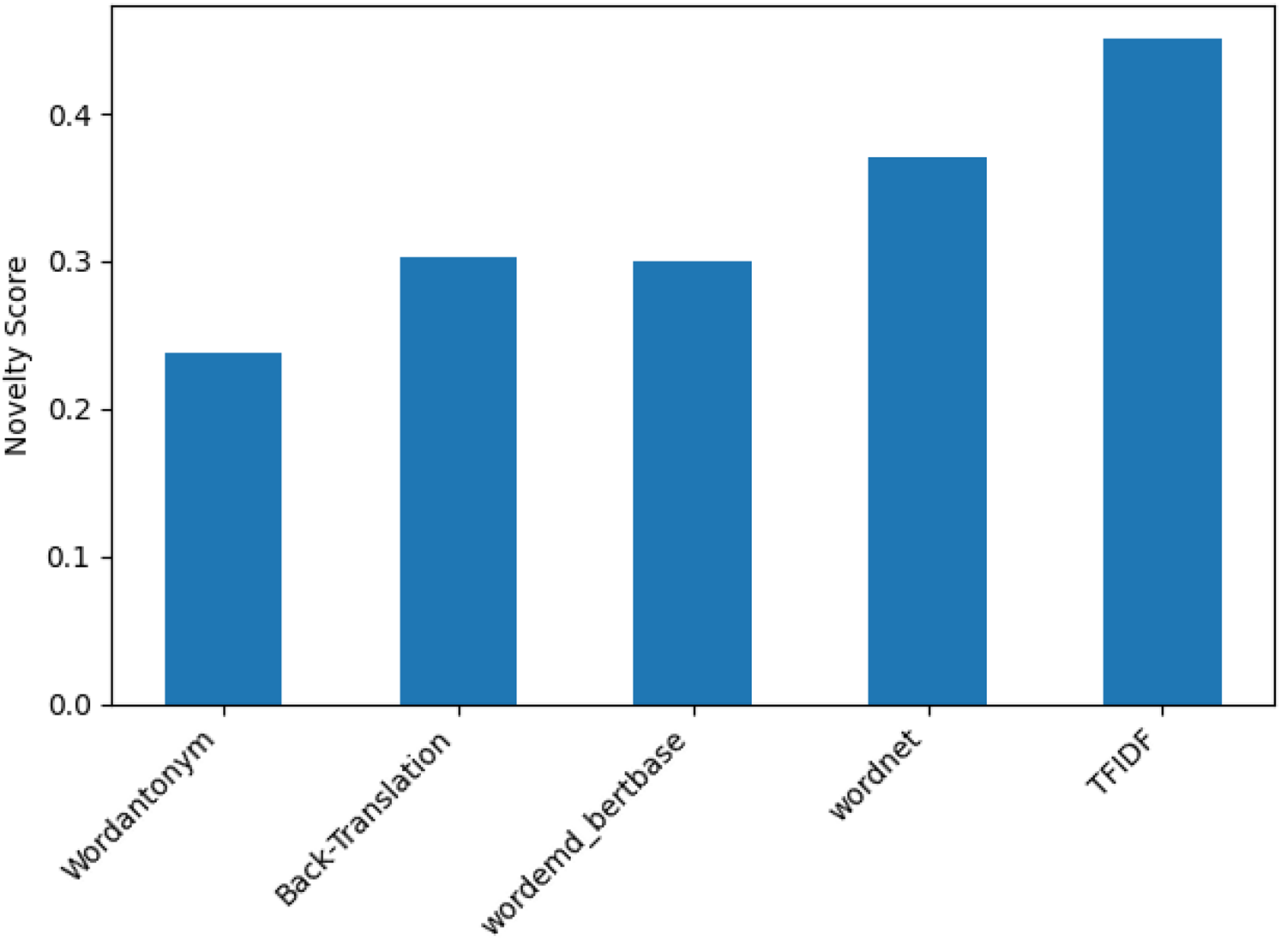
Novelty of Data Generated by DA techniques.

Lexical and Semantic Novelty Scores measure a variation in word choice and meaning from the original text. The balance between novelty and relevance is maintained to eliminate redundant data, ensuring that the enhanced text adds functional variants without altering its meaning or context. Figure 7 summarizes the scores obtained for these metrics; some DA techniques, such as TF-IDF and WordNet, provide new words to the original data, unlike WordAntonym and BackTranslate, which offer fewer new words but ensure preserving the original context or meaning.

Semantic similarity assesses how closely related two pieces of text are in terms of meaning. In contrast, semantic novelty measures the degree to which the definition of a piece of text differs from or is unique to another. The Jaccard similarity metric assesses the degree of uniqueness introduced by augmentation techniques and its potential impact on model performance. Typically, as semantic similarity increases, semantic novelty decreases, and vice versa, due to the trade-off between maintaining meaning and introducing new concepts. A more considerable distance between cosines shown in Fig. 7 indicates more significant semantic dissimilarity and potentially more remarkable novelty. Different augmentation strategies are evaluated based on how well important language, relevant phrases, and logical text structure are retained, assessing the quality of machine-generated translations. TTR and Rouge scores for different applied DA techniques are measured. TTR scores can provide indirect information on the variety of generated text in text augmentation. TTR calculates the ratio of unique words to the total number of words in the text. A low TTR score may indicate a lack of diversity; a higher TTR score means the data contains diverse words. Using the ROUGE score, several characteristics of overlap between enhanced and reference texts are measured by the ROUGE-1, ROUGE-2, and ROUGE-3 scores. ROUGE-1 examines single words, ROUGE-2 examines word pairs or brief phrases, and ROUGE-3 considers the general organization and flow of the text.

**Fig. 8:**
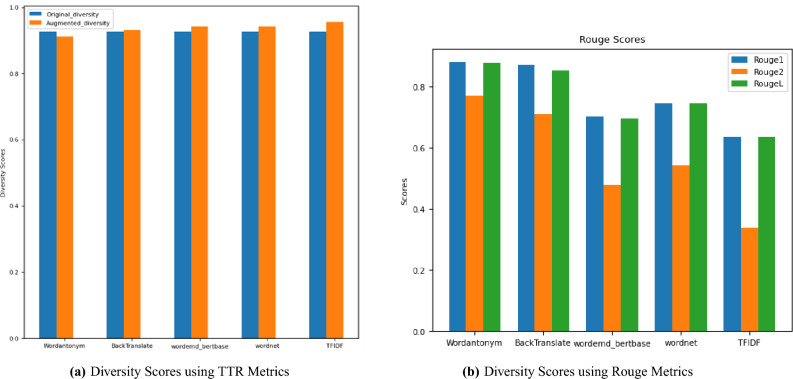
Diversity Scores for Augmentation Techniques.

To achieve the objective of assessing the degree to which the augmented text maintains important details while adding an unexpected element to the data, using both measures, Fig. 8 shows that there is an improvement in diversity after the augmentation process based on TTR and Rouge metrics such as WordAnotym, Backtranslation DA techniques. However, some DA augmentation techniques (WordNet, TF-IDF, and Wordemd_base) decrease the dataset’s diversity because the Rouge measures diversity at the sequence of words. TTR Metrics seem more representative of diversity when focusing on individual words, unlike Rouge metrics, which capture diversity at the n-gram level.

To assess the ability of DA techniques to preserve the original label of the generated data as a vital data quality perspective. We introduce a method for relating the generated data to the original training data. Our technique for measuring the preservation of the label for each DA technique is as follows: Each DA technique assigns a label to the generated text (the predicted label), which matches the label of the original input text. First, we train a baseline classifier (RF) using the labeled original data. Then, we use the trained RF model to test and evaluate the newly generated tweets (as test data) and assign the generated text its proper label. Finally, we compare the classification labels of the baseline (truth labels) to those of the generated text (predicted labels) to measure the accuracy and ability of the DA technique to preserve the label. For example, for all generated text (using the WordAntonym technique) for the actual class, the RF baseline classifier classifies 95% of it as accurate and 5% as fake. So, the WordAntonym technique has the ability 95% to preserve the label of its generated text.

**Fig. 9:**
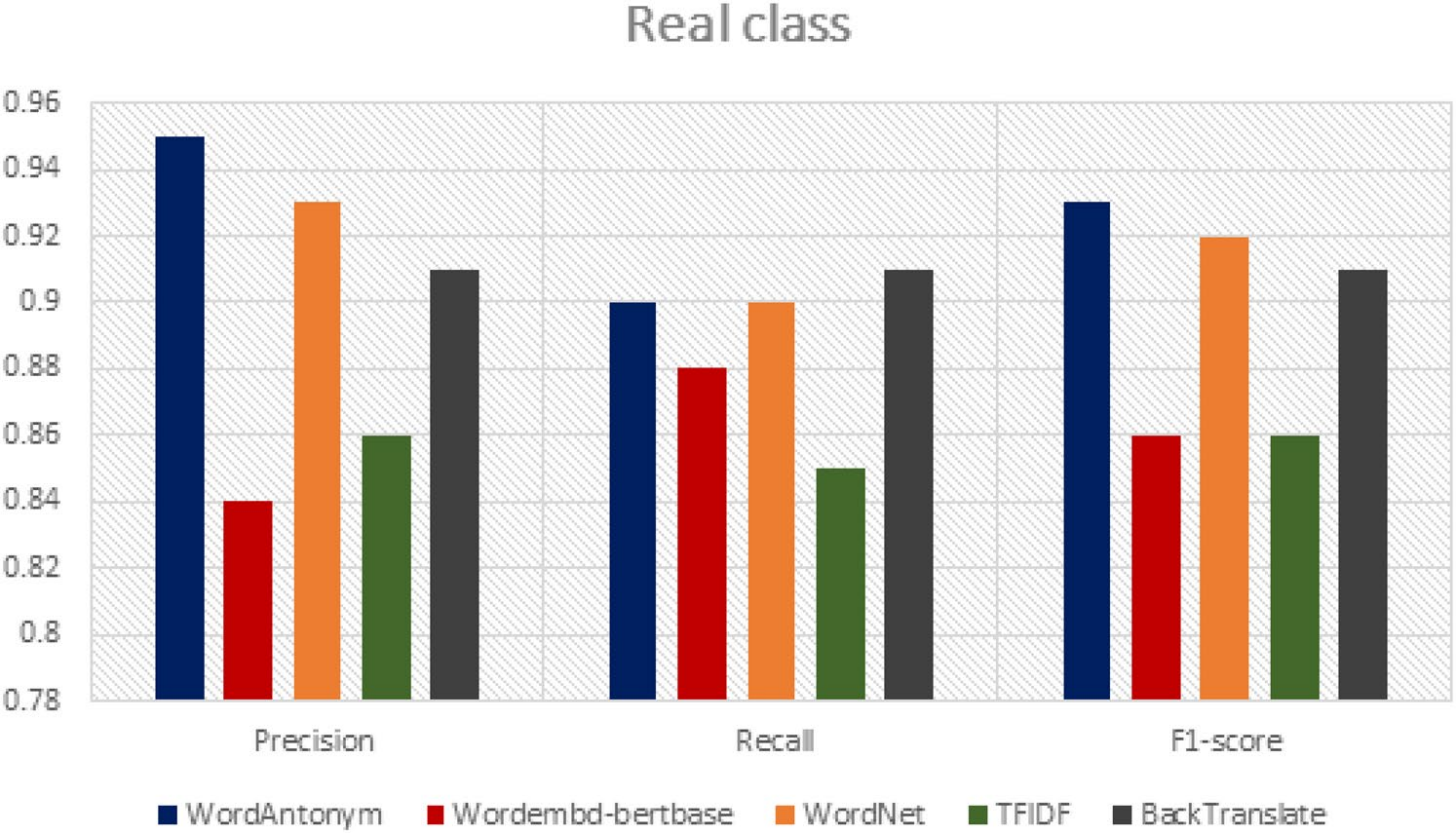
Comparison of DA techniques ability to preserve real class label.

**Fig. 10:**
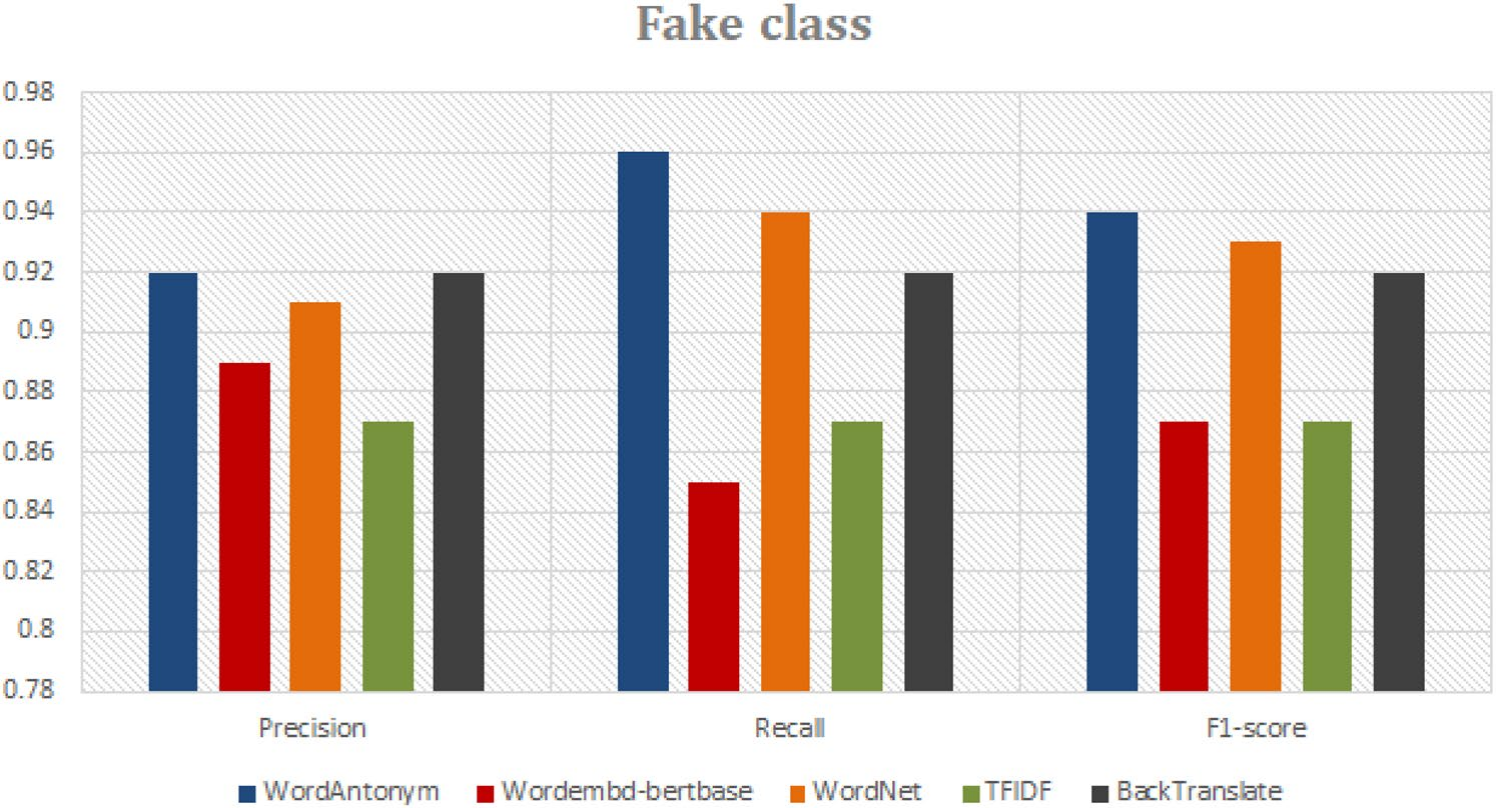
Comparison of DA techniques ability to preserve fake class label.

As shown in Figs. 9 and 10 for real label class, the generated tweets by WordAntonym and Wordnet have the highest precision 95% and 93%. However, regarding recall, Backtranslate surpasses the other DA techniques by 91%. Figure 10 shows the results for fake class labels. It indicates that the tweets generated by WordAntonym and WordNet have a recall of 96% and 94%, respectively, and F1-scores of 94% and 93%. However, Precision, Backtranslate, and WordAntonym surpass the other DA techniques, 92% and 92.1%, respectively. Therefore, we recommend using the three WordAntonym, WordNet, and Backtranslate for augmentation to focus on preserving the label as a data quality measure.

### Experiment D: performance of text classification using ensemble augmentation-based techniques

Machine learning models can be made more robust and general by employing various strategies that produce a range of data variations, thereby improving their ability to generalize to unseen data^43^. Despite this, this strategy has a drawback: The enhanced dataset may become too noisy, making it more difficult for the models to learn. An effective way to manage this variability is to combine several augmentation strategies. However, controlling the diversity of the supplemented data is crucial when using data augmentation techniques. Therefore, it is essential to determine the ideal ratio of noise to diversity in the enhanced dataset. To achieve this balance, a careful assessment of the model’s performance on these datasets and rigorous experiments with different combinations of augmentation approaches are necessary.

**Table 15:** Results of RF with Ensemble Augmentation on Augmented Dataset(No. of new Tweets: 1,203).

Method	Accuracy(%)	Prec.(%)	Recall(%)	F-score(%)
Antonym-Wordnet	88.7	88	89	89
GPT-2	85.5	86	85.7	86
GPT-2- Antonym	90.2	91	90	90
GPT-2- Antonym-WordNet	88.3	89	88	88

**Table 16:** Results of AraBERT with Ensemble Augmentation on Augmented Dataset (No. of new Tweets: 1,203).

Method	Accuracy(%)	Prec.(%)	Recall(%)	F-score(%)
WordAntonym - Wordnet	90.3	90	89.5	90.1
GPT-2	86.6	86.4	86.7	86.7
GPT-2 - Antonym	91.0	90.9	90.8	91
GPT-2 - Antonym - WordNet	90.8	89.5	89	90.5

The results of Experiment A are compared with those of Experiment D. This comparison determines the effectiveness of combining multiple data augmentation (DA) techniques in enhancing model precision and resilience, and it helps understand how each method affects the data augmentation process and the model’s overall performance. After removing the duplicates, the ensemble approach combines the tweets generated by the ensemble techniques such that the total combined tweets are equal to 1203, the same tweets added to the original training data in Experiment A. We compare the performance of models trained on datasets created by various combinations of augmentation techniques, as shown in Tables 15 and 16, including Synonym-WordNet, GPT-Synonym, and GPT-Synonym-WordNet.

Both the RF and AraBERT models achieve the highest accuracy with the GPT −2 antonym technique. The Antonym-WordNet strategy greatly enhances GPT’s performance but remains less effective than the GPT-Antonym strategy in both models. Generally, AraBERT outperforms RF, especially in WordAntonym-WordNet and GPT-Antonym models. AraBERT achieves a maximum accuracy of 91%, while RF achieves 90.2%. From results of Experiment A and D, By comparing the best accuracy of models in Table 6 and Table 7 to Table 15 and Table 16, RF’s accuracy is decreased by 2.25% (92.45% vs. 90.2%), while for AraBERT is decreased by 4.81% (95.81% vs. 91.0%).

We can conclude that ensemble augmentation decreases model performance, while increased diversity through ensemble augmentation can improve generalization. Combining different augmentation techniques can, however, produce excessive noise, leading to overfitting or misclassification. Therefore, carefully selecting and tuning augmentation strategies are crucial to maximize model performance without compromising data quality.

### Comparison between our proposed approach and the previous approaches

The results of our study are presented in Table 17 along with those of earlier methods^19,69^ that used the same dataset. The performance of RF and AraBERT is significantly improved over the earlier methods when combined with our suggested augmentation techniques. Alsudias and Rayson^69^ introduce a dataset of Arabic fake news and apply traditional ML models, such as SVM and LR, for classification; they did not use any text augmentation before the classification task. However, Mohamed et al.^19^ did not introduce the effect of different DA techniques; only the back-translation DA technique is applied. Unlike our work, they did not consider preserving labels, semantic similarity, or novelty issues. Furthermore, the related work section highlights the distinction between our approach and these state-of-the-art methods, as shown in Table 2.

**Table 17:** Comparison of the results of our approach and previous works. AraBERT-Aug/RF-Aug refers to using Random Forest/AraBERT classifier with data augmentation.

Approach	Classifier	Accuracy (%)	Precision (%)	Recall (%)	F-score (%)
Alsudias, L. & Rayson^69^	Logistic Regression	82.24	83.71	72.59	75
Mohamed, E. A. et al.^19^	RF-Aug	81.30	81.02	82.25	81.40
	AraBERT-Aug	95.46	95.52	95.30	94.82
Our Approach	RF-Aug	93.58	94	94	94
	AraBERT-Aug	97.22	97	96	97

## Implication for real-world application

Large language models for Arabic text classification can be developed and implemented using insightful results from the proposed ensemble-based Arabic text augmentation method. The following are some ways that the insights from the results can influence and shape LLM usage in this area:

- Sectors that rely on Arabic healthcare text classification, whether for sentiment analysis, fake news detection, or topic classification, should prioritize Arabic-specific LLMs like AraBERT. This method should produce more accurate classification results since these models take into account the morphological complexity, syntax, and semantics of the Arabic language.
- In sentiment analysis, synonyms are handy for distinguishing between positive and negative sentiments. Accordingly, companies engaged in Arabic NLP should assign resources to enhance data using antonym substitution techniques. Integrating these technologies with LLMs can improve the systems’ ability to handle subtle linguistic variations, resulting in better classification results.
- Although GPT-Antonym-WordNet has lower accuracy (89.5% compared to AraBERT), it processes the largest number of tweets (3,536). Accordingly, the developed model can be applied to situations that require scalability and the ability to handle large volumes of tweets, such as real-time monitoring of major social media sites and analyzing consumer feedback. Combining GPT-based models with innovations like antonyms and WordNet can enable industries to strike a balance between speed and accuracy when processing large volumes of Arabic social media data.
- A stand-alone GPT’s performance is significantly worse than that obtained after adding WordNet and antonyms, at 85.5% and 86.6%, respectively. When developing LLMs for Arabic tweet categorization, businesses should optimize the models by adding domain-specific features, such as incorporating specialized information or utilizing Arabic language resources.

## Conclusion & future work

This study examines how data augmentation affects the performance of Arabic healthcare fake news classification using RF and AraBERT classifiers. A multi-metric analysis, including cosine similarity and BERTScore, is conducted to determine the optimal threshold for comparing the original and augmented Arabic tweets. Additionally, the quality of the augmented data generated through our proposed techniques is evaluated based on diversity, novelty, label preservation, and semantic integrity. The results demonstrate that the AraBERT model outperforms the RF model in every augmentation strategy. Specifically, AraBERT-Aug achieves an accuracy of 97.22%, while RF-Aug achieves an accuracy of 93.58%. With WordAntonym, AraBERT demonstrated superior performance, improving by 2.68%. With WordNet, it improved by 5.62%. Also, BERTScore-based similarity demonstrates more effectiveness than cosine similarity in capturing acceptable semantic variations. By introducing ensemble augmentation, combining WordAntonym and GPT2 yields the highest accuracy compared to other ensemble techniques. However, the ensemble augmentation performance is less than that of individual DA techniques. Key performance metrics, including the F1 score, cosine similarity, BERTScore, and Jaccard similarity, offer valuable insights into how data augmentation improves the model’s ability to identify fake news. Future research could investigate how sophisticated models, such as GPT-4, enhance Arabic data augmentation methods. Moreover, integrating explainable AI techniques may increase the transparency and dependability of the model, while incorporating meta-learning and semi-supervised learning approaches may enhance the model’s flexibility.

## Data Availability

Arabic healthcare data related to COVID-19 is used from December 2019 to April 2020, which were included in the data set used in https://aclanthology.org/2020.nlpcovid19-acl.16.
